# *In Vitro* evaluation of the anti-pancreatic cancer activity of *epimedium* herb

**DOI:** 10.3389/fphar.2024.1389221

**Published:** 2024-07-01

**Authors:** Yangfeng Chen, Han Xia, Xiaohong Zhong

**Affiliations:** ^1^ College of Horticulture, Hunan Agricultural University, Changsha, China; ^2^ Changsha Central Hospital, Changsha, China

**Keywords:** pancreatic cancer, *epimedium*, hyperoside, icariin, baohuoside I, interleukin-4, interleukin-13

## Abstract

**Introduction:** Pancreatic cancer (PC) is a particularly aggressive malignancy with limited therapeutic options. The search for innovative treatments has focused on traditional Chinese medicine, specifically *epimedium*. This research investigates *epimedium*’s active ingredients, potential targets, and underlying mechanisms in treating PC.

**Methods:** High-performance liquid chromatography (HPLC) was used to quantify the active components of *epimedium* and HPLC-Q-TOF-MS was employed for qualitative identification. Potential targets of *epimedium*’s active ingredients were identified using the TCMSP, ETCM, CTD, and Swiss Target Prediction databases. Potential PC-related targets were sourced from DisGeNET, GeneCards, and OMIM databases. A Venn diagram was utilized to identify overlapping PC-related and *epimedium* targets. Core targets and pathways were elucidated through protein-protein interaction (PPI) network analysis, Gene Ontology (GO) assessments, and Reactome pathway enrichment analyses. Molecular docking techniques investigated interactions between active compounds and these targets. The expression and prognostic implications of target genes were evaluated using GEPIA2 and the Human Protein Atlas (HPA) databases. *In vitro* studies assessed the impact of *epimedium* extract (EPE) on Panc-1 cell viability, and Western blot analysis examined the expression levels of key targets.

**Results:** Network pharmacological indicate that *epimedium* econtains active components such as baohuoside I, icariin, hyperoside, and epimedin B, which have potential therapeutic effects against PC. *In vitro* assays confirmed that EPE significantly reduced the viability of Panc-1 cells. Western blot analysis revealed a considerable decrease in the expression of key targets in EPE-treated cells, including AKT1, EGFR, p-EGFR, JUN, BCL2, IL6, and SRC. The R-HSA-1280215: Interleukin-4 and Interleukin-13 signaling pathways involving these genes were identified as potential therapeutic targets.

**Discussion:** *Epimedium* holds promise as a candidate for treating PC. The modulation of interleukin-4 and interleukin-13 signaling pathways could be a pivotal mechanism by which *epimedium* impedes tumor development. Further research is warranted to validate these findings and explore the clinical applicability of *epimedium* in PC treatment.

## Introduction

Pancreatic cancer (PC) is a highly aggressive malignancy that has been on the rise over the past decade, with its incidence increasing from 43,140 to 60,430 cases (Jemal et al., 2010; Siegel et al., 2021). As a result, it is now the third leading cause of cancer-related deaths in the United States, after lung and breast cancers (Grossberg et al., 2020). While established risk factors for PC include age, gender, race/ethnicity, family history of the disease, smoking habits, diabetes, and obesity (Yamaguchi et al., 2017), its prevalence is still increasing due to the aging global population (Goral, 2015; Jia et al., 2018; Klein, 2021). Despite this alarming trend, there is still hope for the development of new treatments and therapies to combat this deadly disease. PC is typically asymptomatic at onset and progresses rapidly with early metastasis, resulting in many patients being diagnosed too late for surgery and a high mortality rate (Song et al., 2020; Ye et al., 2021).

Surgery with negative margin resection under a microscope is challenging and only benefits a few patients (Kamisawa et al., 2016). Therefore, radiotherapy or chemotherapy is necessary for most patients. In recent years, targeted therapies and immunotherapies have emerged as novel treatments, offering more options for those with advanced disease. However, they can be associated with adverse effects such as rash, liver dysfunction, or diarrhea (Rudin et al., 2008; Suzumura et al., 2012), which can affect the patient’s quality of life or lead to discontinuation of therapy. Furthermore, these costly drugs are effective only for certain patients, highlighting the need to find more effective yet less toxic new drugs.

Chinese herbal medicine has a long history of use in the treatment of critically ill patients. Traditional Chinese herbs have been used for centuries to treat various ailments and diseases, including cancer (Zhang et al., 2014; Zhang et al., 2016). However, it is essential to note that some whole herbs may also contain toxins and non-medicinal chemicals. Further research is needed to understand the mechanisms and pathways involved in cancer treatment. Investigating active substances with precise chemical structures separated from herbs may be beneficial. Many natural compounds, such as paclitaxel and vinblastine, have been found to possess anticancer activity, either directly or indirectly. Even at high concentrations, multiple natural chemicals can be effectively tolerated by patients (Jiang et al., 2016; Rejhová et al., 2018). Thus, novel natural substances offer great promise for developing effective yet less toxic anticancer drugs.

*Epimedium*, guided by Traditional Chinese Medicine (TCM) principles of reinforcing positive and eliminating negative energy, employed in cancer treatment for an extensive period (Liu et al., 2021; Zhao et al., 2022). The polysaccharide (EPS) derived from the *epimedium* plant has been demonstrated to enhance the secretion of immunomodulatory cytokines by peritoneal macrophages and T cells, thereby reducing tumor burden. Moreover, icariin, a derivative of *epimedium* known as a prenylflavonoid, has demonstrated antitumor effects on diverse human cell lines, including breast, prostate, endometrial cancer, renal cell carcinoma, and leukemia (Guo et al., 2011; Tong et al., 2011). Furthermore, Wang et al. (2019) discovered its capability to inhibit tumor cell growth by generating reactive oxygen species and inducing DNA damage.

In 2007, Hopkins and colleagues introduced the concept of “network pharmacology,” which involves investigating the effects of drugs on disease networks. This approach established a “drug-target-disease” network, offering an innovative approach to drug development. This approach enabled researchers to understand better the complex interactions among drugs, targets, and diseases while identifying new drug targets and potential drug combinations. Through the aid of network pharmacology, researchers can now enhance their ability to predict the efficacy and safety of drugs and develop new drugs that are more effective and possess fewer side effects (Liu and Du, 2009). In recent times, network pharmacology has been successfully employed in numerous studies to investigate the mechanisms of TCM (Wu et al., 2020). To advance our understanding of the mechanisms and pathways involved in the therapeutic effects of *epimedium* on PC, we applied this approach to examine its impact on PC and confirm its regulatory associations with key signaling pathways.

## Materials and methods

### Cells and reagents

*Epimedium* was obtained from Hunan Zirantang Traditional Chinese Medicine Pieces Co., Ltd. (production batch number: 230401). Hyperoside, Epimedin A, epimedin B, epimedin C, icariin, and baohuoside I standards (Purity ≥98%) were purchased from Shanghai Yuanye Co., Ltd. (Shanghai, China). The human pancreatic cancer cell line PANC-1 was acquired from Procell Co., Ltd. (Wuhan, China). DEME medium (#C11995500CP), 10% fetal bovine serum (#FSP500), and penicillin-streptomycin were obtained from Gibco. The ELISA kit was purchased from BOSTER Technology Co., Ltd. (Wuhan, China). DMSO, CCK-8 kit, RIPA lysis buffer (#PC0020), and BCA protein assay kit (#PC0020) were purchased from Solarbio Technology Co., Ltd. (Beijing, China). The antibodies used are as follows: EGFR (#ab52894), p-EGFR (#ab40815), AKT1 (#ab81283), JUN (#ab32385), IL6 (#ab214429), SRC (#ab109381), BCL2 (#ab182858) and β-actin (#ab8227) were purchased from Abcam (Cambridge, United Kingdom) and Cell Signaling Technology (Boston, MA, United States).

### Preparation of *epimedium* extract

*Epimedium sagittatum* (Sieb.etZucc.) Maxim., a Berberidaceae plant, was purchased from Hunan Zirantang Traditional Chinese Medicine Pieces Co., Ltd. (production batch number: 230401) and identified by Professor Xiaohong Zhong. *Epimedium* extract (EPE) preparation method involved taking 100 g of *epimedium*, adding 8 times 70% ethanol, soaking for 30 min, boiling for 1.5 h, centrifuging at 10,000 RPM for 30 min, and collecting the supernatant. This extraction process was repeated twice, and the supernatants were mixed and evaporated to obtain a powder and stored at −20°C for later use.

### Chemical determination of extract contents

Flavonoids are regarded as the main active ingredient in *epimed*ium (Yang et al., 2020; Zhao et al., 2018). Flavonoids were detected and identified, and the contents were determined by liquid chromatography-ultraviolet/quadrupole-time-of-flight mass spectrometry (LC-Q-TOF-MS) (Wang et al., 2010; Zhao et al., 2018).

The preparation of the reference solution was carried out as follows: Accurately weigh an appropriate amount of each reference substance (hyperoside, epimedin A, epimedin B, epimedin C, icariin, baohuoside I) (5–10 mg) into a 50 mL volumetric flask, add 50% methanol to dissolve and dilute to the mark, filtered through a 0.45 μm microporous membrane. Preparation of test solution: epimedium extract was accurately weighed and placed in a conical flask with a stopper. 70% ethanol (20 mL) was added, the weight was accurately measured, and the sample was sonicated for 10 min. The samples were then removed and left to cool down to room temperature, and the loss in weight was made up with 70% ethanol. After filtering through a 0.45 μm microporous membrane, the filtrate was used as the test solution.

In brief, an ultra-performance liquid chromatograph, Agilent 1290, coupled with an Agilent 6495 tandem mass spectrometer (Agilent Technologies, Wilmington, DE, United States) was used. The chromatographic conditions were as follows: The chromatographic column was Hypersil BDS C18 (250 mm × 4.6 mm, 5 μm), column temperature was 25°C. The mobile phases of HPLC were composed of acetonitrile (A) and water (B). The linear gradient was adopted as follows: 24%–26% A at 0–20 min, 26%–30% A at 20–30 min, 30%–45% A at 30–45 min, 45%–60% A at 45–50 min, 60% to 24%A at 50–60 min. The flow rate was 1.0 mL/min with detection at 270 nm. The injection volume was 10.0 μL. MS conditions: electrospray ion source (ESI), positive and negative ion modes, and scanning range of m/z 100–2,000; dry gas temperature 350°C, dry gas flow rate 10 L·min^−1^, spray voltage 30 psi, fragmentor voltage 135 V, cone voltage 65 V, ion spray voltage (IS) 3500 V (positive ion mode)/-4000 V (negative ion mode). For MS/MS analyses, auto-MS/MS was performed with a collision energy between 10 and 55 eV.

### Screening of related targets

The potential targets of active ingredients of epimedium were screened from the following databases: Traditional Chinese Medicine Systems Pharmacology (TCMSP) database (https://www.tcmsp-e.com/) (Ye et al., 2022) Encyclopedia of Traditional Chinese Medicine (ETCM, http://www.tcmip.cn/ETCM2/front/#/) (Xu et al., 2019), Swiss Target Prediction (http://www.swisstargetprediction.ch/), Comparative Toxicogenomics Database (CTD) (https://ctdbase.org/). Ultimately, the potential targets of *epimedium* were identified by standardizing and removing duplicates using the Uniprot database (https://www.uniprot.org/).

### Acquisition of potential anti-pancreatic cancer targets and their shared targets with *epimedium*

To identify genes associated with pancreatic cancer, we conducted a thorough search of the GeneCards, GeneCards, and OMIM databases, focusing on the “*Homo sapiens*” species and using the keywords “pancreatic cancer.” As a result, we were able to pinpoint genes that have been previously reported to be linked with pancreatic cancer. Subsequently, we carefully eliminated any duplicate entries and false positives from the gene list, cross-referencing it with previously identified potential targets of *epimedium*. Consequently, we successfully identified potential targets for combating pancreatic cancer using *epimedium*.

### Protein-protein interaction construction

By inputting the potential anti-pancreatic cancer targets of *epimedium* into the STRING database (https://cn.string-db.org/), restricted to the “*H. sapiens*” species and filtered with a minimum interactome requirement score of >0.7 (Von Mering et al., 2005), we acquired protein-protein interaction relationships. Subsequently, we employed Cytoscape (version 3.7.1) to visually represent the PPI network. Moreover, we installed the CytoNCA package to conduct network topology analysis, aiming to identify crucial target genes associated with the mechanism of icariin. In order to identify highly interconnected subnetworks within the PPI network, we employed Cytoscape’s Molecular Complex Detection technique (MCODE) (Bader and Hogue, 2003).

### Protein functional enrichment analysis

To gain a deeper understanding of the potential biological functions of *epimedium* in the treatment of pancreatic cancer, we carried out gene ontology (GO), biological processes (BPs), and Reactome pathway enrichment analyses using the DAVID Database (Sherman et al., 2022) and Reactome Database (Wright et al., 2022). The resulting data was organized according to their adjusted *p* values.

### Ingredient-target-pathway network construction

An Ingredient-Target-Pathway (I-T-P) network was established in Cytoscape (version 3.7.1), encompassing overlapping genes, pathways, and selected active components. Nodes of diverse colors represented various clusters, while edges delineated node relationships.

### Molecular docking verification of core active ingredients and core targets

Molecular docking was performed to validate the interaction between the core active ingredient and the core target identified in the PPI network. The three-dimensional structures of the core targets were obtained from the Protein Data Bank (PDB, www.rcsb.org), and the two-dimensional structures of the top five core compounds were acquired from PubChem in SDF format. PyMOL v2.4, an open-source molecular graphics tool, was used to remove solvent and organic components to prepare for molecular docking. Subsequently, AutoDock Tools (version 1.5.6) were employed for molecular docking, and the resulting data was analyzed and interpreted using PyMOL v2.4.

### Validation of core targets in GEPIA2 and HPA database

The Gene Expression Profiling Interactive Analysis (GEPIA 2; http://gepia2.cancer-pku.cn/) web server is a publicly available database that analyzes gene expression in tumor and normal samples from TCGA and GTEx. We used GEPIA two to analyze the expression levels of core targets relevant to our research question in normal pancreatic and pancreatic adenocarcinoma (PAAD) tissues. Additionally, we investigated the overall survival data of these core targets using the Human Protein Atlas (HPA; https://www.proteinatlas.org/), an open-access database that employs various omics technologies. HPA provides comprehensive mappings of human protein expression patterns in cells, tissues, and organs through antibody-based imaging, mass spectrometry-based proteomics, and transcriptomics.

### Experimental validation

#### Cell culture and viability assays

Cell culture and viability assays were performed as described previously (Shen et al., 2020). Briefly, Panc-1 cells were cultured in 10% fetal calf serum and 100 U/mol penicillin-streptomycin solution at 37°C, 5% CO_2_, and 95% relative humidity. After the intervention, each cell was in the logarithmic growth phase. EPE was dissolved in DMSO at a concentration of 200 mg/mL and then diluted to different concentrations of EPE (0, 100, 200, 300, 400, 500, and 600 μg/mL) for pretreatment. The pretreatment time was 48 h. Cell viability was assessed using a Thermo Fisher microplate spectrophotometer with the CCK-8 assay, following the manufacturer’s instructions. The optical density (OD) of the solution at 450 nm in each well was measured. All data were normalized to control wells without cells and expressed as mean ± standard deviation (SD).

#### Western blot

Panc-1 cells were seeded in 6-well plates at a density of 3 × 10^5^ cells per well. After incubating for 48 h, EPE (200 μg/mL or 400 μg/mL) was added and treated for 48 h. The cells were then collected, and total protein was extracted using radioimmunoprecipitation assay (RIPA) buffer, which contained phenylmethylsulfonyl fluoride (PMSF), aprotinin, and phosphatase inhibitors. The supernatant was collected after centrifugation (14,000 × *g*, 4°C for 15 min), and the protein concentration was determined using a BCA protein assay kit (Genview, United States). 25 μg of each protein sample was separated by SDS-PAGE and transferred to a polyvinylidene fluoride (PVDF) membrane (Millipore, Bedford, MA, United States). The membranes were then blocked with 5% bovine serum albumin (BSA) for 2 h at room temperature. Primary antibodies for AKT1, EGFR, p-EGFR, JUN, BCL2, IL6, and SRC were added and incubated overnight at 4°C. After three washes, an enzyme-labeled secondary antibody IgG (1:2000) was used to incubate for 2 h at room temperature under dark conditions. Finally, a secondary antibody (1:5000, ab clone) was added and incubated at room temperature for 2 h. Finally, membranes were washed and developed by adding enhanced chemiluminescence (ECL) substrate (Thermo Fisher Scientific, Rockford, United States). Proteins were visualized using the intelligent gel imaging system iBright FL1000 (Thermofisher, Rockford, United States).

#### Statistical analyses

Statistical analysis was conducted on SPSS 22.0 (SPSS, Chicago, IL, United States), with a data-processing method of independent-sample *t*-test. Comparisons between groups were statistically analyzed, and *p < 0.05, **p < 0.01, ***p < 0.001, ****p < 0.0001 was considered to be statistically significant.

## Results

### Identification and content of active ingredients in *epimedium*

All the calibration curves showed a good linear regression relationship in the test range (r > 0.999). RSDs of precision, stability, and repeatability were all less than 3.0%. Therefore, the HPLC method was precise, accurate, and sensitive enough for simultaneous quantitative analysis of the six flavonoids in epimedium extract. The HPLC analysis identified hyperoside, epimedin A, epimedin B, epimedin C, icariin, and baohuoside I as the primary compounds, with EPE at 200 μg/mL containing 1.6 μg/mL of hyperoside, 5.8 μg/mL of icariin, 5.9 μg/mL of epimedin A, 20.3 μg/mL of epimedin B, and 3.4 μg/mL of epimedin C, 4.8 μg/mL of baohuoside I.

**FIGURE 1:**
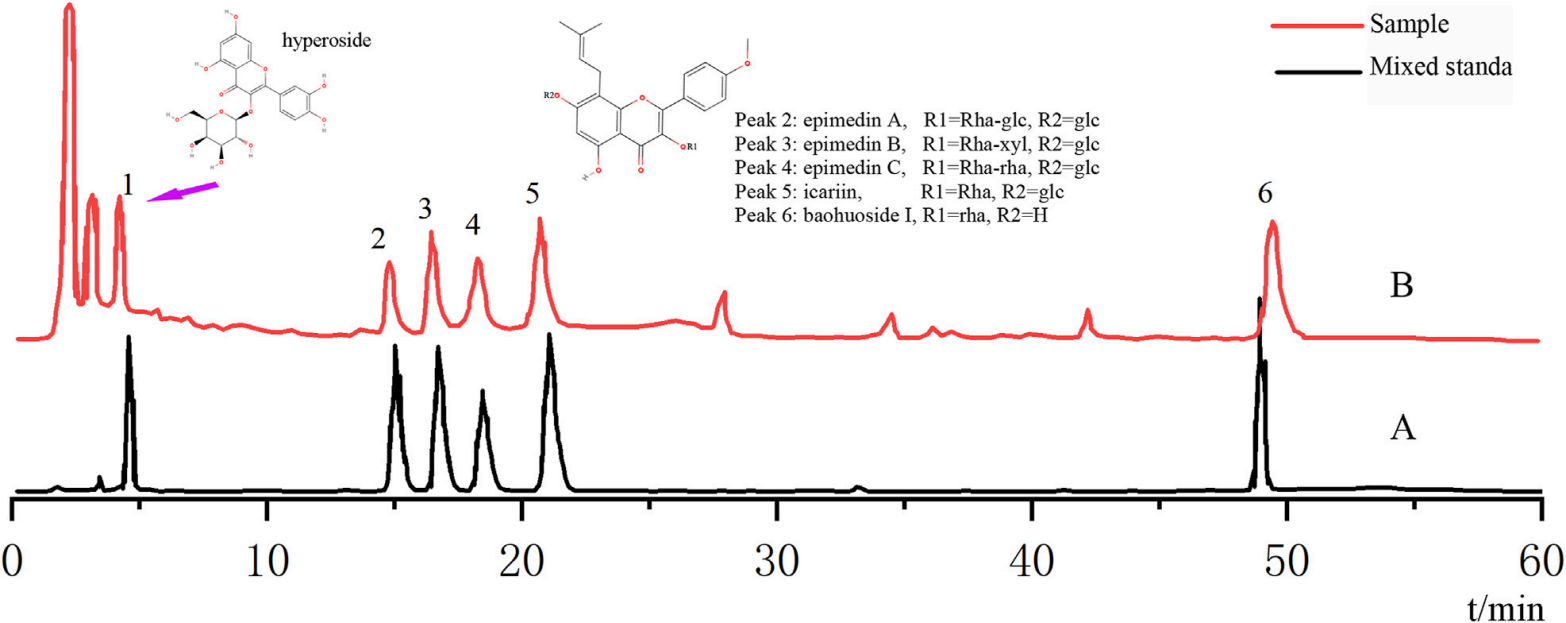
Analysis of the chemical composition of *epimedium* extract by HPLC. **(A)** HPLC chromatogram of mixed standard; **(B)** HPLC chromatogram of the sample.

Based on the optimization of the elution program, we used high-performance HPLC-Q-TOF-MS/MS to perform a qualitative analysis of compounds in epimedium extract. By comparing the retention time (t_R_) and characteristic fragment ions with reference compounds and literature data, a total of six compounds were identified. See Figure 1 and Table 1 for details.

**TABLE 1:** Retention time (t_R_), MS data, and UV spectra for identifying six compounds in *epimedium* extract by HPLC-Q-TOF-MS/MS.

Peak no.	R1	R2	tR (min)	Identification	Formula for M	[M-H]-		References
						Measured mass	Calcd mass	
1	---	---	4.67	Hyperoside	C_21_H_20_O_12_	463.0875	463.0882	Zhao et al. (2018)
2	Rha-glc	glc	14.38	Epimedin A	C_39_H_50_O_20_	837.2825	837.2823	Wang et al. (2010), Zhao et al. (2018)
3	Rha-xyl	glc	16.93	Epimedin B	C_38_H_48_O_19_	807.2714	807.2717	Wang et al. (2010), Zhao et al. (2018)
4	Rha-rha	glc	18.76	Epimedin C	C_39_H_50_O_19_	821.2866	821.2874	Wang et al. (2010)
5	Rha	glc	23.16	Icariin	C_33_H_40_O_15_	675.2291	675.2283	Wang et al. (2010), Zhao et al. (2018)
6	Rha	H	49.88	Baohuoside I	C_27_H_30_O_10_	513.1753	513.1766	Zhao et al. (2018)

### Acquisition of potential targets of *epimedium* against PC

Based on TCMSP, ETCM, CTD database, and Swiss Target Prediction, a total of 174 potential targets were identified for the six active ingredients from *epimedium*, including hyperoside, epimedin A, epimedin B, and epimedin C, icariin, and baohuoside I (Supplementary Table S1).

**FIGURE 2:**
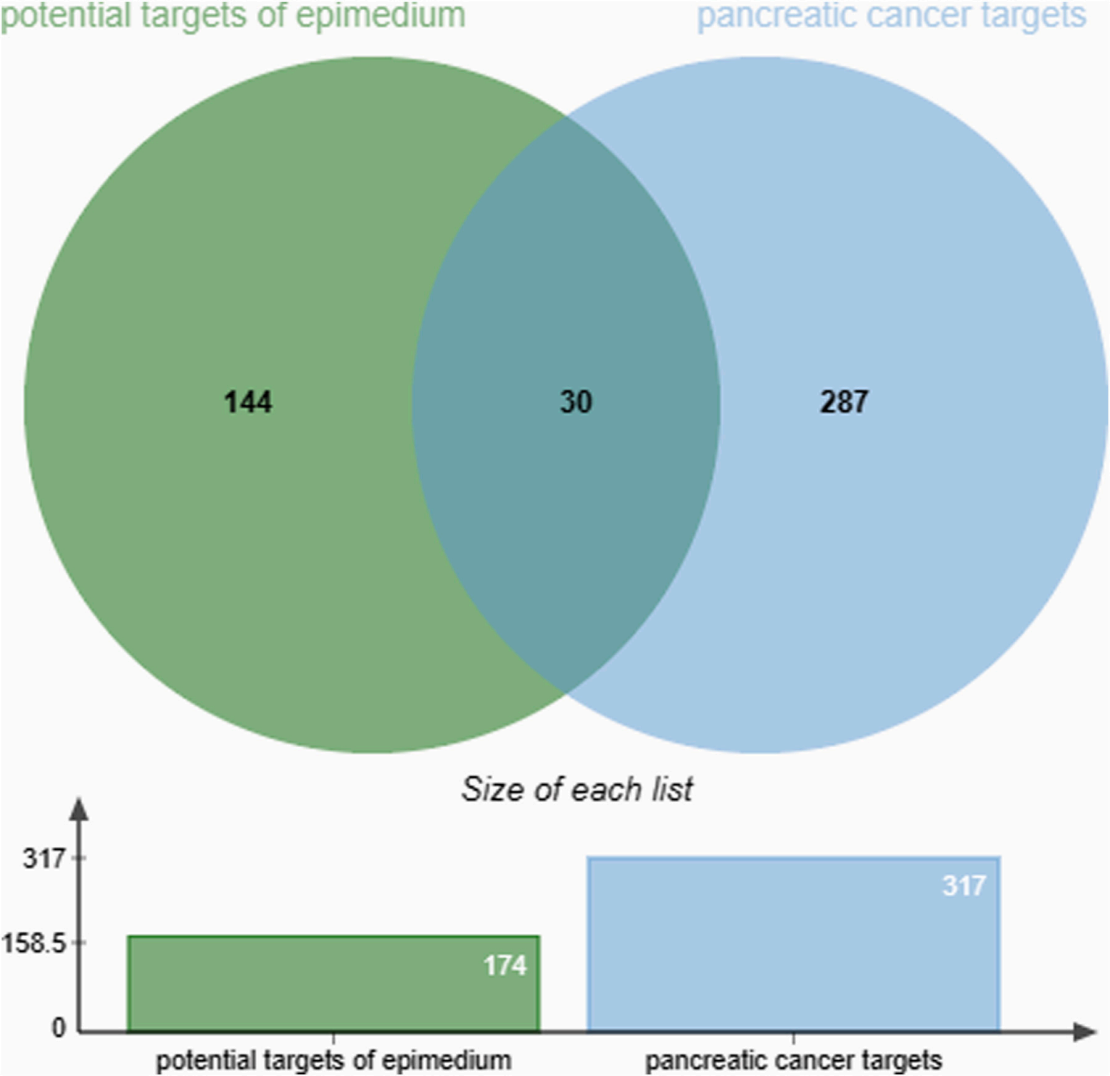
The intersection of the active ingredient and pancreatic cancer targets is represented in 30 overlapping targets.

The GeneCards, GeneCards, and OMIM databases revealed 316 targets associated with PC. Subsequently, a Venn diagram was utilized to identify 30 overlapping targets between the PC-related targets and the potential targets of *epimedium* (Figure 2; Table 2).

**TABLE 2:** The overlapping targets of active ingredient targets and Pancreatic cancer targets.

Uniprot ID	Gene Symbol	Uniprot ID	Gene Symbol
O14746	TERT	P12931	SRC
O43521	BCL2L11	P15056	BRAF
P00533	EGFR	P15692	VEGFA
P01133	EGF	P17174	GOT1
P01137	TGFB1	P27361	MAPK3
P01375	TNF	P28482	MAPK1
P01584	IL1B	P31749	AKT1
P03372	ESR1	P37231	PPARG
P05231	IL6	P42345	MTOR
P05412	JUN	P60568	IL2
P07477	PRSS1	Q07812	BAX
P07900	HSP90AA1	Q13485	SMAD4
P10275	AR	Q14790	CASP8
P10415	BCL2	Q15465	SHH
P11388	TOP2A	Q16236	NFE2L2

### Construction of a protein-protein network

**FIGURE 3:**
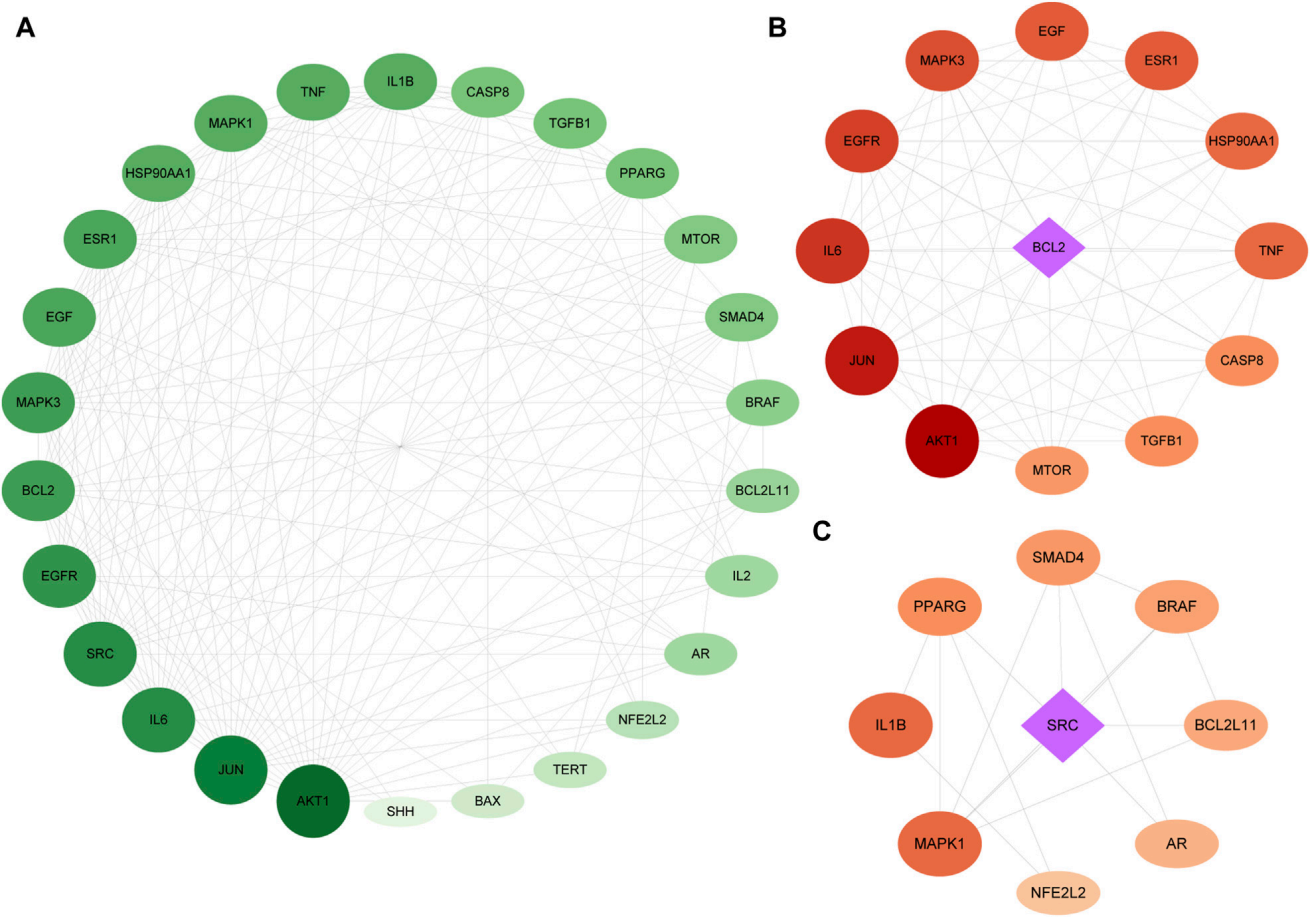
PPI of the intersection targets. The node’s size indicates the degree of the target protein in the network. Panel **(A)** is a PPI network diagram arranged according to a degree, with two clusters detected in the *epimedium*-anti-PC PPI network. Panels **(B,C)** show clusters 1 and 2, respectively, with diamonds representing the seed nodes of each cluster.

The genes related to the 30 overlapping targets were used to establish protein-protein interaction (PPI) relationships through the String database. The resultant PPI network was visualized using Cytoscape 3.7.1, revealing 26 protein nodes and 168 edges representing their interactions. Nodes with a deeper shade of red indicated higher degree values, signifying a strong association between these targets and PC (Figure 3A).

We utilized MCODE analysis to identify three functionally related protein clusters based on topology within the PPI network. The cluster attributes are presented in Figures 3B, C. In PPI networks, clusters formed by interlinked nodes often represent protein complexes or pathways. Similarly, clusters in protein similarity networks typically indicate protein families, where the proteins within a cluster exhibit similar structures and functions.

### GO enrichment and KEGG enrichment

**FIGURE 4:**
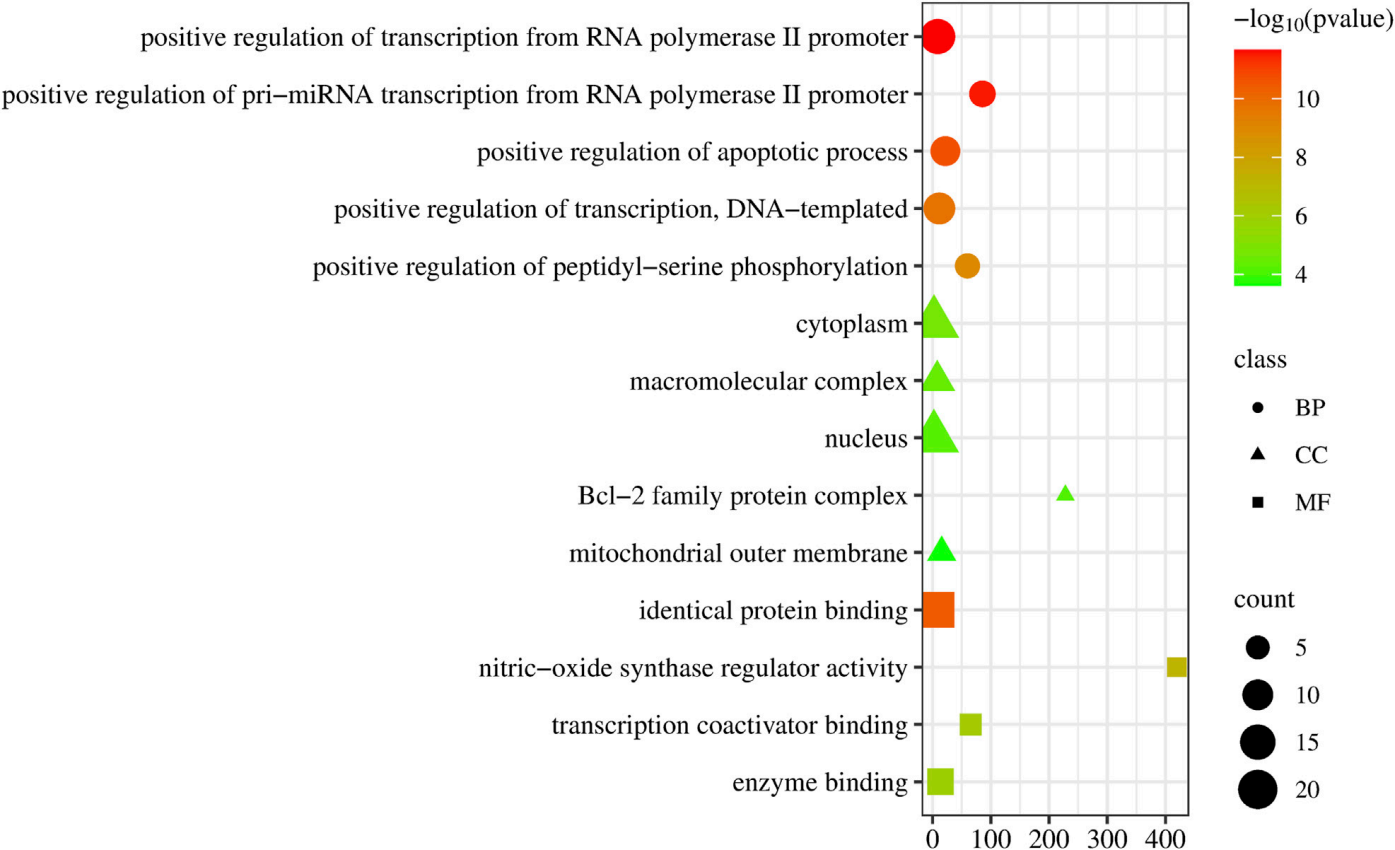
Dot plot of the GO enrichment of *epimedium* against pancreatic cancer. The horizontal axis represents the enrichment rate of the input genes in the GO Term, while the vertical axis represents the GO term name. The color scale indicates different *p*-value thresholds, and the dot size indicates the number of genes corresponding to each term.

Upon uploading the 30 overlapped targets to the DAVID database for GO enrichment analysis, we identified 129 biological processes (BP). The top five processes included positive regulation of transcription from RNA polymerase II promoter, positive regulation of pri-miRNA transcription from RNA polymerase II promoter, positive regulation of apoptotic process, positive regulation of transcription, DNA-templated, positive regulation of peptidyl-serine phosphorylation. Additionally, 32 cellular components (CC) were identified, with the top five being cytoplasm, macromolecular complex, nucleus, Bcl-2 family protein complex, and mitochondrial outer membrane. Moreover, 32 molecular functions (MF) were identified, with the top five being identical protein binding, nitric-oxide synthase regulator activity, nitric-oxide synthase regulator activity, transcription coactivator binding, and enzyme binding (Figure 4).

**FIGURE 5:**
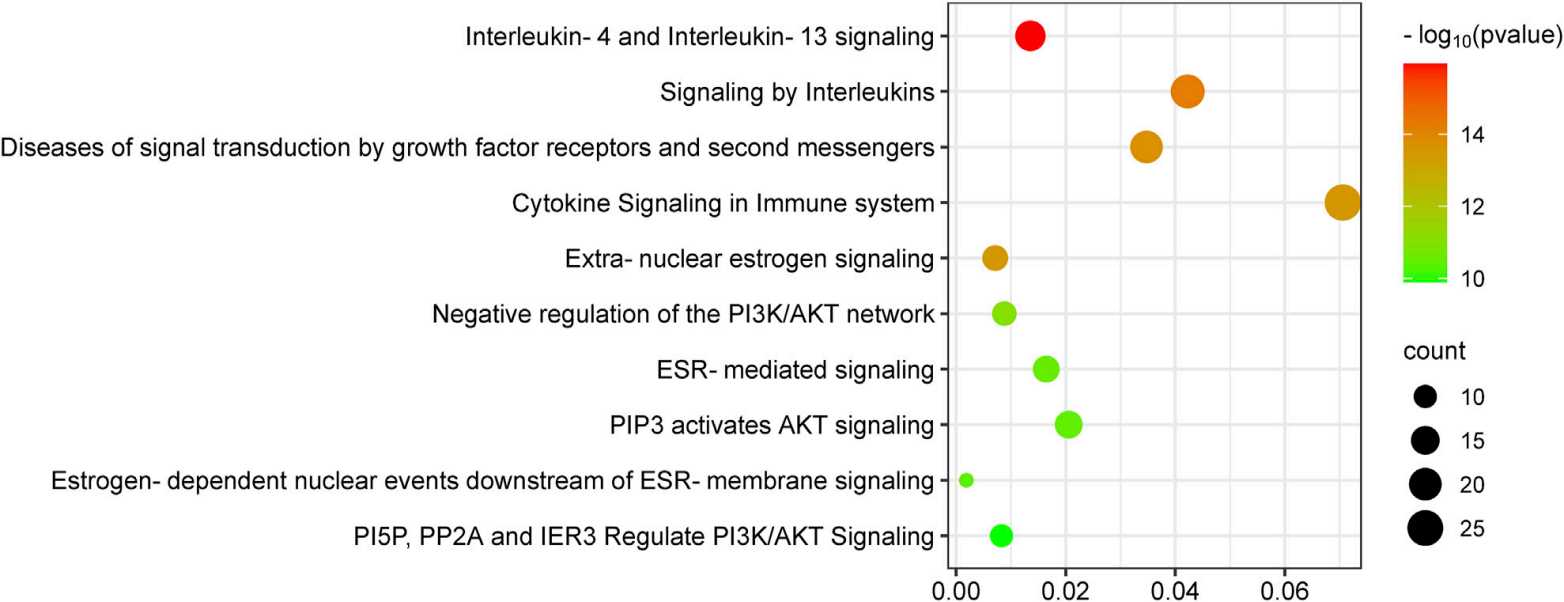
KEGG enrichment analysis of target genes of *epimedium* against pancreatic cancer. The number of genes enriched in each KEGG pathway term is shown in different colors, showing the circle size and the *p*-value.

The Reactome pathway enrichment analysis identified 657 pathways, with the top 10 pathways visually represented in Figure 5. These pathways exhibit notable enrichment in processes associated with anti-PC effects, encompassing Interleukin-4 and Interleukin-13 signaling, Signaling by Interleukins, Diseases of signal transduction by growth factor receptors and second messengers, Cytokine Signaling in Immune system, and Extra-nuclear estrogen signaling.

### Ingredient-target-pathway network construction

**FIGURE 6:**
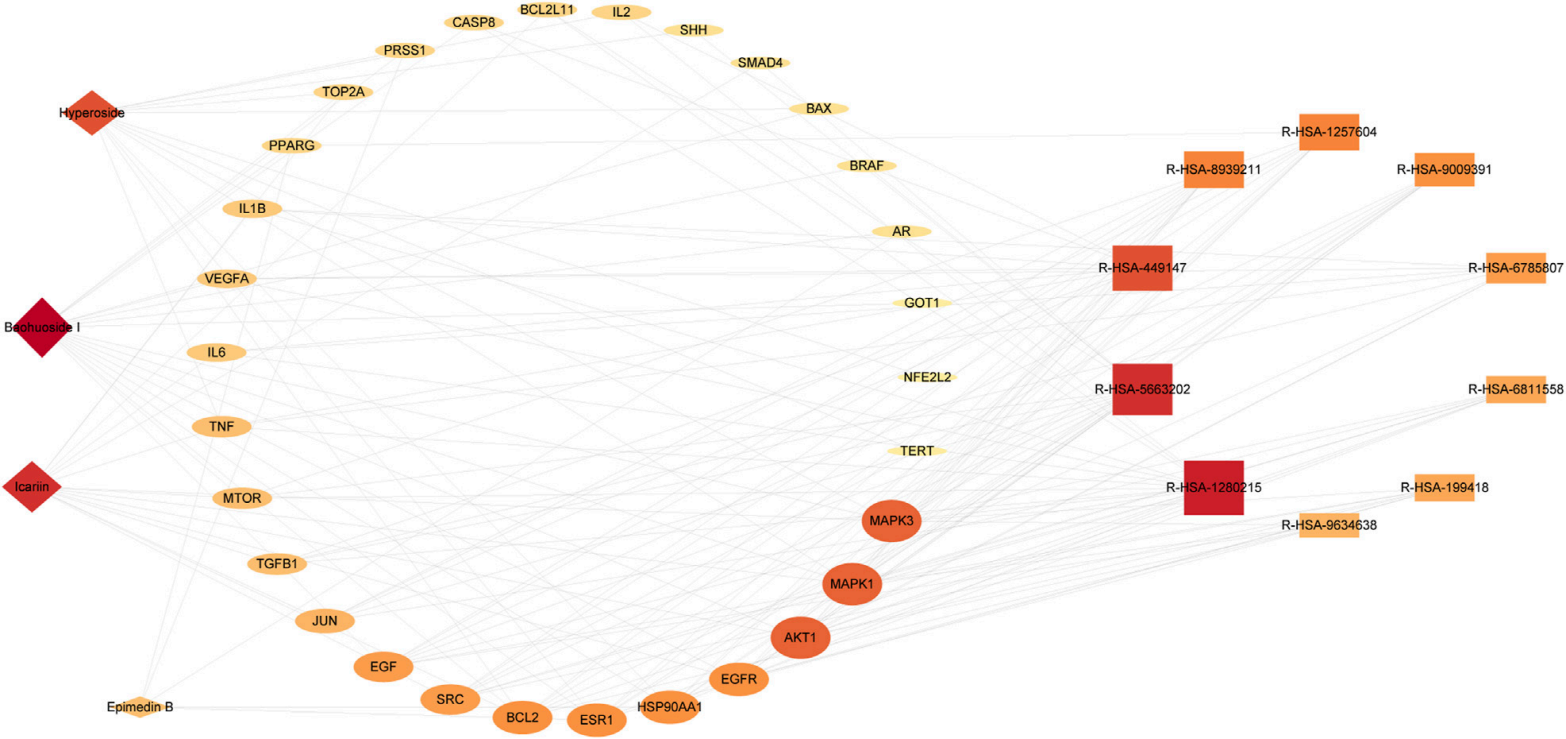
Ingredient-target-pathway network diagram. The active ingredient is displayed as a diamond shape, the targets as an ellipse, and the pathway as a rectangle.

The analysis of the Ingredients-Targets-Pathways network revealed 44 nodes and 152 edges, consisting of four active constituents of *epimedium* (baohuoside I, icariin, hyperoside, epimedin B), 30 potential anti-PC targets, and 10 pathways (Figure 6). Our findings illustrated that baohuoside I had the highest level of connectivity with 18 targets, followed by icariin (15), hyperoside (13), and epimedin B (5), indicating their potential involvement in regulating pathways associated with PC.

### Molecular docking verification of core active ingredients and core protein targets

The results of molecular docking studies have revealed the binding strength of the essential active constituents present in *epimedium* with their specific protein targets. After conducting I-T-P network screening, we identified baohuoside I, icariin, hyperoside, and epimedin B as the core compounds. The hub protein targets comprise the top five node degree proteins (AKT1, JUN, IL6, SRC, and EGFR) and two seed nodes in clusters (SRC and BCL2).

**FIGURE 7:**
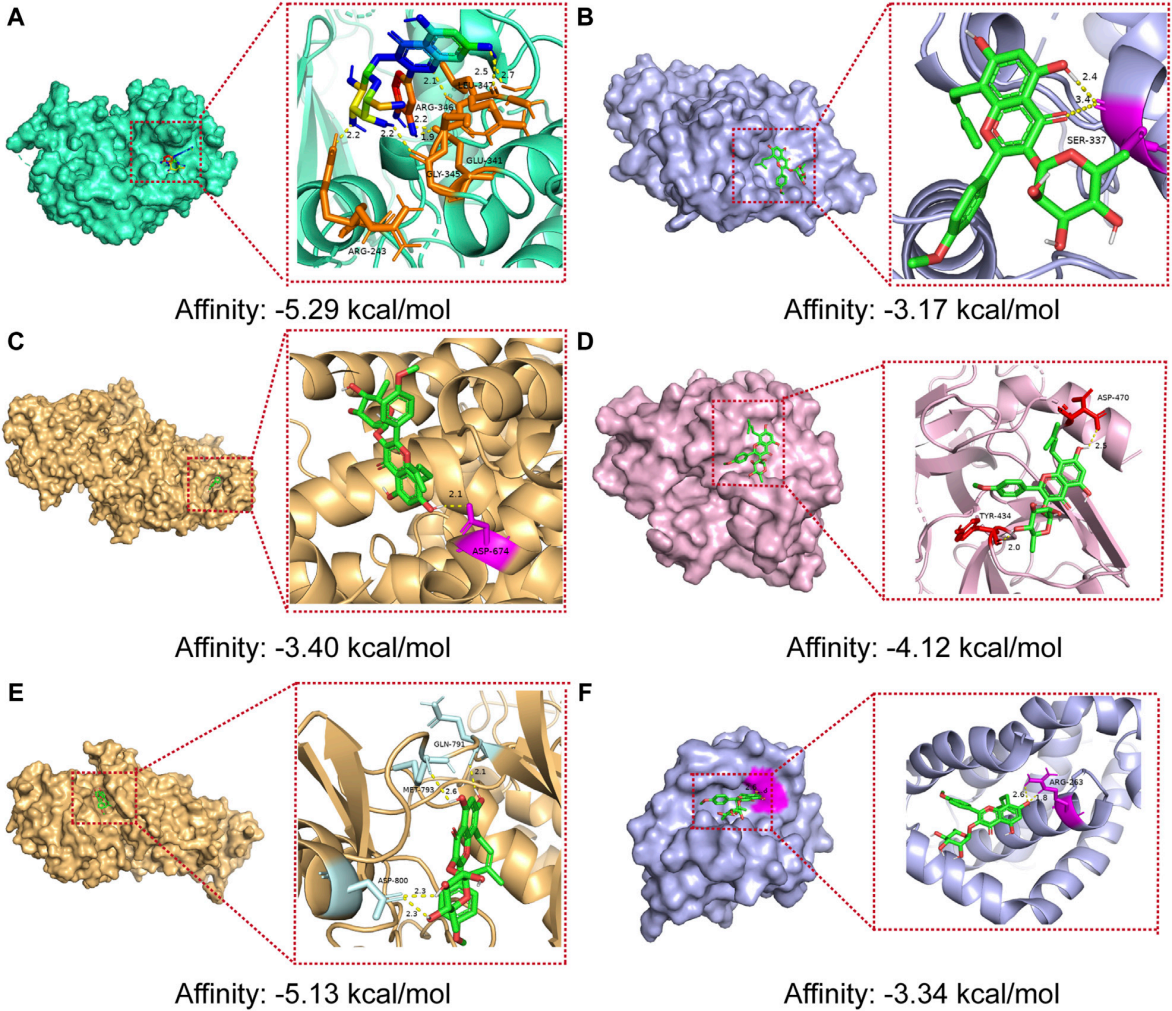
Molecular docking models of core compounds and core protein targets **(A)** AKT1 - Hyperoside, **(B)** JUN - Baohuoside I, **(C)** IL6 - Baohuoside_I, **(D)** SRC - Baohuoside _I, **(E)** EGFR - Baohuoside _I, and **(F)** BCL2 - Baohuoside I.

The binding affinities of the six core active ingredients to their respective target proteins were investigated. Baohuoside I displayed solid binding affinities, ranging from −3.17 kcal/mol to −5.13 kcal/mol, with JUN, IL6, SRC, EGFR, and BCL2, respectively, while icariin and hyperoside exhibited a high affinity of −4.63 and −5.29 kcal/mol with AKT1. The strong binding of the small-molecule active ingredients was attributed to various interactions (as shown in Figure 7; Table 3).

**TABLE 3:** The binding energy values of core compounds of *epimedium* and core targets.

Target	Compounds	Binding affinity/(kcal/mol)
AKT1 (4GV1)	Baohuoside I	−4.61
	Icariin	−4.63
	Hyperoside	−5.29
	Epimedin B	−2.68
JUN (2P33)	Baohuoside I	−3.17
	Icariin	−1.79
	Hyperoside	−2.02
	Epimedin B	−1.45
IL6 (5SFK)	Baohuoside I	−3.40
	Icariin	−1.22
	Hyperoside	−1.84
	Epimedin B	−1.82
SRC (3SOS)	Baohuoside I	−4.12
	Icariin	−2.51
	Hyperoside	−2.78
	Epimedin B	−2.62
EGFR (3IKA)	Baohuoside I	−5.13
	Icariin	−2.84
	Hyperoside	−3.91
	Epimedin B	−1.25
BCL2 (6U64)	Baohuoside I	−3.34
	Icariin	−2.54
	Hyperoside	−2.18
	Epimedin B	−1.05

### Validation of key targets in GEPIA2 and HPA database

**FIGURE 8:**
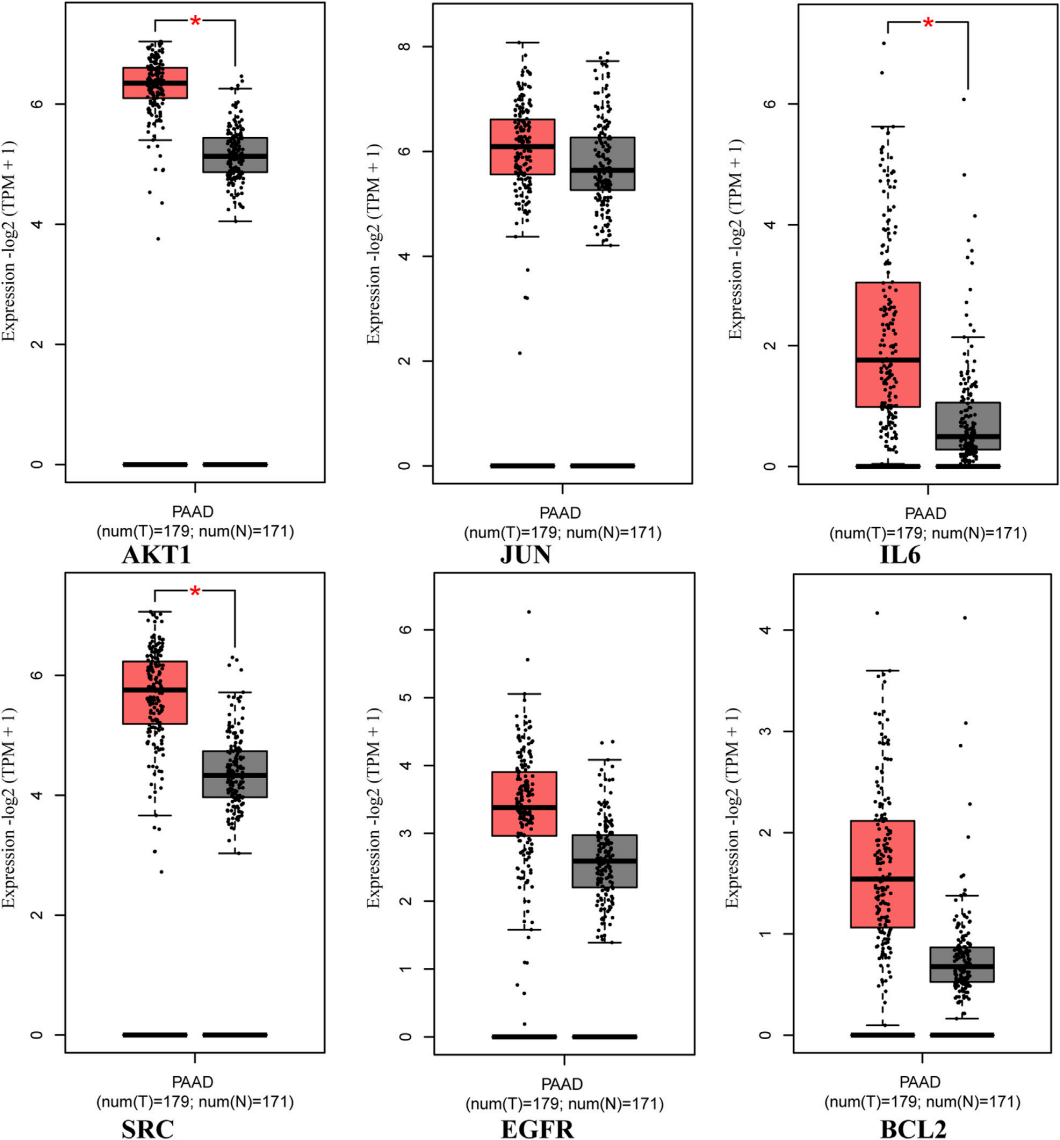
Expression of core targets in the GEPIA two database. The red box plots represent the tumor, and the gray box plots represent normal. Match TCGA normal and GTEx data.

**FIGURE 9:**
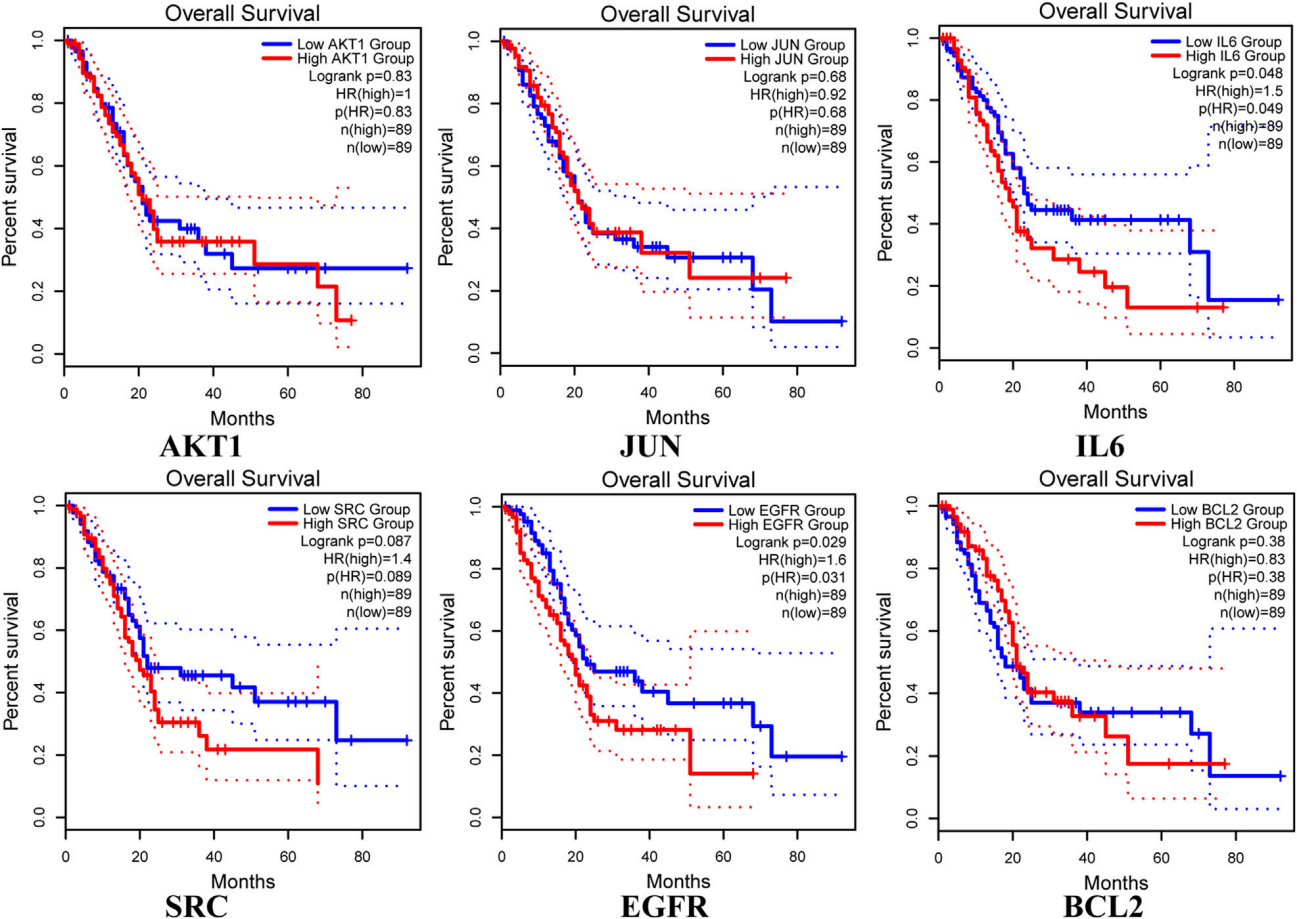
Overall survival of hub genes in the HPA database, with blue representing low expressions and red representing high expressions. Kaplan-Meier survival curves were independently plotted for each gene.

The analysis using the GEPIA two database revealed that the expression of core targets (AKT1, IL6, and SRC) was higher in PAAD samples compared to normal pancreas tissues (*p* < 0.05; Figure 8). Moreover, the examination of overall survival data from the HPA database indicated that high expression of EGFR and IL6 was correlated with a significantly poorer prognosis (*p* < 0.05; Figure 9).

### *In vitro* experimental validation

**FIGURE 10:**
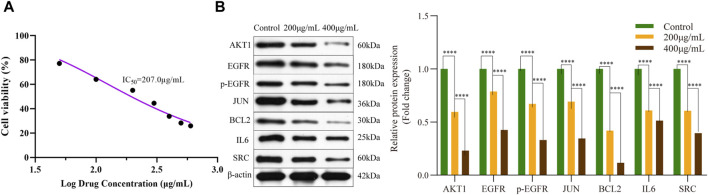
*Epimedium* extract suppressed R-HSA-1280215: Interleu in-4 and Interleukin-13 signaling pathway in PANC-1. **(A)** Cell viability of PANC-1 cells treated with different concentrations of *epimedium* extract; **(B)** Effect of AKT1, EGFR, p-EGFR, JUN, BCL2, IL6 and SRC expression in PANC-1 cells analyzed by Western blot (****p < 0.0001); IC_50_, half maximal inhibitory concentration.

To investigate the impact of EPE on pancreatic cancer cells, we employed Cell Counting Kit-8 (CCK-8) to assess the cell viability of Panc-1 cells following treatment with varying concentrations of EPE (Figure 10). Our findings revealed a noteworthy effect of EPE on the viability of Panc-1 cells in a dose-dependent manner with a half-maximal inhibitory concentration (IC_50_) of 207.0 μg/mL.

The R-HSA-1280215: Interleu in-4 and Interleukin-13 signaling was the first pathway obtained in the KEGG pathway enrichment analysis, involving downstream proteins. To verify the effect of EPE on key targets, we utilized the Western blot method to measure the expression of AKT1, EGFR, p-EGFR, JUN, BCL2, IL6 and SRC proteins in Panc-1 cells. The results, shown in Figure 10B, indicate a significant decrease in the expression of AKT1, EGFR, p-EGFR, JUN, BCL2, IL6, and SRC in EPE-treated Panc-1 cells.

## Discussion

PC exhibits a high mortality rate and is frequently diagnosed at advanced stages, posing significant challenges for effective treatment. Therefore, it is essential to develop novel therapeutics and drugs to improve the quality of life and extend the lifespan of affected individuals. The progression of PC involves intricate biological processes, such as metabolic disorders, local inflammation, and aberrant molecular pathways (Motoo et al., 2011; Ansari et al., 2019). As a result, single-drug therapies targeting a single pathway may not be sufficient to achieve optimal therapeutic efficacy (Li et al., 2019).

Studies have indicated that various components of TCM, including the Qingyi Huaji formula (Hua et al., 2014; Zhang et al., 2013) and Huang-Qin-Tang (Saif et al., 2014), can effectively boost the immune system and suppress the proliferation of PC cells. These components play a beneficial role in enhancing immunity against infectious diseases and mitigating factors that disrupt normal physiological functions (Li et al., 2015). Moreover, single-herb TCM or herbal formulas have demonstrated the ability to engage multiple targets and pathways of action.

In this study, network pharmacology was employed to investigate the mechanism of *epimedium*’s action against PC. By analyzing the PPI network, we identified six potential therapeutic core targets (AKT1, EGFR, JUN, BCL2, IL6, and SRC) that exhibited significant correlations with PC. Subsequently, we performed molecular docking calculations to evaluate the binding affinities of *epimedium*’s active ingredients to these proteins. The results revealed that baohuoside I, icariin, hyperoside, and epimedin B displayed high binding affinities to AKT1, EGFR, JUN, BCL2, IL6 and SRC, indicating their potential relevance in PC treatment.

The validation of key targets using the GEPIA2 and HPA databases revealed significantly higher expressions of AKT1, IL6, and SRC in PAAD tissues compared to normal pancreatic tissues. Furthermore, high expressions of EGFR and IL6 were associated with significantly poorer survival rates.

In the development and progression of cancer, AKT1, JUN, IL6, SRC, EGFR and BCL2 plays a critical role. Akt1 (serine-threonine protein kinase) is a part of the PI3K-dependent signaling pathway and is a key cell survival protein functionally involved in antiapoptosis in various cancers (Goswami et al., 2006). Epidermal growth factor receptor (EGFR) is a trans-membrane receptor tyrosine kinase. It plays a critical oncogenic role in cancer growth, survival, migration, and invasion (Jiang et al., 2017). The study demonstrated that epimedium extract inhibits the expression and phosphorylation of AKT1 and EGFR in the pancreatic cancer cell line PANC-1. Similar findings have been previously documented. For instance, Geng et al. (2014) reported that icariside II inhibits the proliferation of transplantable tumors and the EGFR/mTOR signaling pathway, leading to reduced phosphorylation of EGFR and AKT1 proteins. In addition, Ding et al. (2018) indicated that Icariin enhances the sexual function of male mice by activating the PI3K/Akt/eNos/No signaling pathway. Furthermore, Li et al. (2020) demonstrated that icariin, via the EGFR/PI3K/mTOR signaling pathways, decreases the expression and phosphorylation of EGFR and Akt proteins. Moreover, Sun et al. (2022) found that icariin interacts with the estrogen receptor on the cell membrane, resulting in elevated phosphorylation levels of Akt and Creb proteins and increased transcription of genes encoding steroidogenic enzymes and testosterone synthesis.

EGFR^Y1068^ phosphorylation can be characterized by its association with the recruitment of specific signaling proteins and its potential role as a predictive biomarker for treatment response in certain cancer patients (Sette et al., 2015; Wirth et al., 2023). EGFR^Y1068^ phosphorylation induces the recruitment of Grb2 and Gab1, leading to the activation of the AKT and STAT3/5 signaling pathways (Wirth et al., 2023). Prior research indicates that Icariin suppresses the epithelial-mesenchymal transition process induced by epidermal growth factor by downregulating the PI3K/Akt signaling pathway (Wang et al., 2021). Icariin can diminish the expression of ERα36 and hinder E2β-triggered Akt phosphorylation, showing heightened efficacy in suppressing TNBC cell proliferation and facilitating apoptosis when combined with the EGFR inhibitor Cetuximab (Yin et al., 2020). Additionally, it inhibits the activation of ERK1/2 and Akt induced by ionizing radiation (Hong et al., 2013), diminishes STAT3, and Akt in MDSCs, leading to the differentiation of MDSCs into dendritic cells and macrophages (Zhou et al., 2011). In our study, epimedium extract was found to inhibit EGFR^Y1068^ phosphorylation in the pancreatic cancer cell line, PANC-1. However, it should be noted that the epimedium extract used in this study consisted of various components, including hyperoside, epimedin A, epimedin B, epimedin C, icariin, and baohuoside I. Further investigation is required to discern the specific active ingredients and elucidate the underlying mechanisms responsible for the inhibitory effects of epimedium extract on EGFR^Y1068^ phosphorylation.

BCL2 is widely recognized as a multifunctional anti-apoptotic protein in various types of cancer. The overexpression of the BCL2 is linked to tumor initiation, progression, and resistance to current anticancer therapies (Das et al., 2020). Icariin has been shown to enhance its antioxidant and anti-apoptotic functions by inhibiting the phosphorylation of BCL2 (Zhang et al., 2016). Additionally, hyperoside relieved H2O2-induced oxidative stress and attenuated H_2_O_2_-induced apoptosis of granulosa cells, resulting in decreased expression of the BCL2 protein (Wang et al., 2019). Icariin and hyperoside are critical active components of epimedium extract in our study.

## Conclusion

This study employed bioinformatics, network pharmacology, database verification, and *in vitro* validation to identify potential active compounds and pathways of *epimedium* in the treatment of PC. Through the regulation of genes such as AKT1, EGFR, JUN, BCL2, IL6 and SRC, *epimedium* was found to modulate the R-HSA-1280215: Interleu in-4 and Interleukin-13 signaling pathway. The findings suggest that further investigation of *epimedium* as a subsequent treatment for PC is warranted.

## Data Availability

The original contributions presented in the study are included in the article/Supplementary Material, further inquiries can be directed to the corresponding authors.
